# Conserved genotype-independent rhizobacteria promote maize growth

**DOI:** 10.1038/s41522-025-00895-4

**Published:** 2025-12-31

**Authors:** Junnan Fang, Guoliang Wang, Chun Zhang, Guiming Liu, Jiacan Xu, Yuqian Gao, Yajie Guo, Xuming Wang, Tianlei Qiu

**Affiliations:** 1 Beijing Key Laboratory of Agricultural Genetic Resources and Biotechnology, Institute of Biotechnology, Beijing Academy of Agriculture and Forestry Sciences, Beijing, PR China; 2 College of Landscape and Ecological Engineering, Hebei University of Engineering, Hebei, PR China; 3 College of Agronomy, Hebei Sub-center of National Maize Improvement Center of China, State Key Laboratory of North China Crop Improvement and Regulation, Hebei Agricultural University, Hebei, PR China

**Keywords:** Ecology, Ecology, Microbiology

## Abstract

Rhizosphere microbiomes play an essential role in promoting plant growth and health. Although host genotype is known to shape rhizosphere microbial communities, it remains unclear whether core microbial taxa can persist across genetically diverse hosts and contribute to plant performance. Here, we conducted a large-scale analysis of 1005 rhizosphere samples from 335 maize populations to investigate the effects of host genetic variation on rhizosphere microbiota. We observed significant genotype-dependent variation in both bacterial and fungal community diversity and composition. However, community assembly was predominantly governed by stochastic processes, suggesting an evolutionary conservation of rhizosphere microbiota across genotypes. Based on the hypothesis that core microbes may consistently associate with maize independent of genotypes, we identified a core bacterial taxon, ASV245 (*Pseudomonas* sp.), which was consistently enriched across all maize genotypes. The corresponding strain, designated as WY16, was isolated from maize roots and significantly promoted both stem and root growth by activating maize hormone signaling pathways. These findings highlight the persistence and functional roles of genotype-independent core microbes, deepening our understanding of plant-microbiome interactions and providing new insights for microbiome-based strategies in sustainable agriculture.

## Introduction

Maize (*Zea mays* L.) is one of the most important crops globally, serving as food, feed, and fuel. It plays a vital role in ensuring both national food and seed source security^1^. However, global climate change and the degradation of arable land are posing serious challenges to maize production, including stagnating yields and growing pressures from pests and diseases^2^. Traditional strategies relying on chemical fertilizers and pesticides are inadequate for the demands of sustainable agriculture^3,4^. As a result, there is an urgent need for innovative and environmentally friendly solutions in agricultural biotechnology. Research on the plant rhizosphere microbiome offers promising new strategies to address these challenges^4–8^.

The rhizosphere, the soil zone surrounding plant roots, harbors diverse and abundant microbial communities. These microbiomes play essential roles in plant growth and health as the “second genome” of plants^5,9^. They can enhance nutrient uptake, promote plant growth, alleviate environmental stress, and boost immunity^10^. For example, when plants face pathogen attack, they can recruit beneficial microbes via specific signaling pathways to suppress disease. These beneficial microbes not only offer immediate defense but also increase disease resistance in subsequent plant generations^11,12^. Under nutrient deficiency, maize roots selectively recruit rhizobacteria involved in nutrient transformation, improving nutrient cycling and nitrogen acquisition^13^. Research on these beneficial rhizobacteria have identified 25 core bacterial taxa inhabiting maize xylem, contributing to nitrogen-fixing capabilities^14^. Besides, parallel research has revealed that these rhizobacteria can successfully colonize root tissues and subsequently translocate to stems, enhancing maize resistance to stalk rot^15^. Plant growth-promoting rhizobacteria (PGPR) also produce phytohormones such as indole-3-acetic acid (IAA), cytokinin, ethylene, gibberellins, and abscisic acid. These compounds influence endogenous hormone levels or signaling pathways, promoting root development by increasing lateral root formation, root hair density, and root system length^16,17^. However, it remains challenging to accurately identify how specific rhizosphere microbes affect plant physiology and how to use these microbes to enhance crop productivity.

Importantly, plant genotypes significantly influence rhizosphere microbial communities, suggesting complex regulatory interactions between plant genotypes and their associated microbiomes^18,19^. For instance, genetic variation in sorghum has been shown to influence rhizosphere bacterial composition^7^. Analysis combined genome-wide association studies (GWAS), microbiome-wide association studies (MWAS), and microbiome genome-wide association studies (mGWAS) across 827 millet accessions revealed that plant genotype explains only part of the observed phenotypic variation. Furthermore, 257 microbial markers were identified that exhibited significant associations with six agronomic traits and showed that microbial effects were dependent on host genotype^20^. Similarly, analysis of three switchgrass field populations revealed that over half of the core microbial taxa in 1961 switchgrass root samples were strongly associated with host genotype, independent of environmental location^21^. These findings highlight the role of the microbiome in shaping plant phenotypes and offer new strategies for integrating microbiome insights into traditional plant breeding.

Recent studies have shown that host genotype can influence rhizosphere microbial community^18,19^. However, it remains unclear which microbial taxa can persist independently of host genetic variation, and the ecological mechanisms underlying the stability of such conserved taxa are poorly understood. A deeper understanding of how host genomes influence rhizosphere microbiomes is essential for the effective application of beneficial microbes in agriculture. Therefore, in this study, we focused on investigating the relationship between maize genotypes and their associated rhizosphere microbial communities. Specifically, we aimed to identify microbial features that are conserved across diverse maize genotypes and to clarify the links between host genotype and microbial function. We analyzed 1005 rhizosphere microbiome samples from 335 maize populations representing four genotype groups using high-throughput sequencing. We characterized the diversity, composition, and assembly patterns of both bacterial and fungal communities in the rhizosphere. Network analysis and prevalence-abundance framework were then applied to identify core microbial taxa. To further validate their importance, we isolated representative core microbes and conducted functional assays. We further explored how stochastic community assembly processes contribute to the persistence of these conserved taxa, focusing on *Pseudomonas* as a representative PGPR. This integrative approach provides new insight into the mechanisms that maintain functionally important microbial members across host genotypes and advances our understanding of the ecological basis of plant-microbiome interaction. This study addresses three key questions: (1) How does maize genotype regulate the assembly and structure of rhizosphere microbial communities? (2) What ecological and functional mechanisms support the persistence of core beneficial microbes across diverse genotypes? (3) Can conserved core microbes with genotype-independent growth-promoting effects be identified and potentially applied to enhance maize productivity?

## Results

### Rhizosphere bacterial and fungal community diversity and composition among maize genotypes

**Fig. 1:**
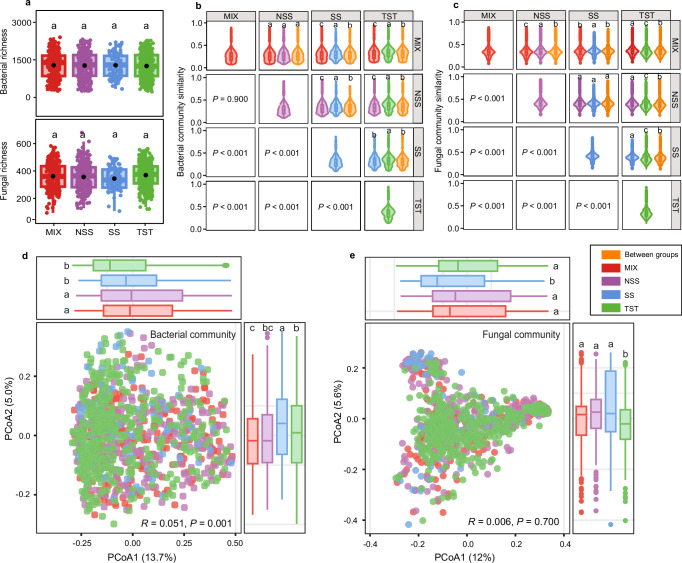
Distinct rhizosphere bacterial and fungal communities among four genotype maize plants. **a** The bacterial and fungal richness of four genotype maize. Comparison of (**b**) bacterial and (c) fungal community similarities within and between any two genotype groups. Principal coordinate analysis (PCoA) based on Bray-Curtis distance for (**d**) bacterial and (**e**) fungal communities among four genotypes. Maize genotypes: MIX, Mixed Subpopulation; NSS, Non-Stiff Stalk; SS, Stiff Stalk; TST, Tropical/Subtropical.

We collected 1005 rhizosphere samples from 335 maize varieties representing four major genotypes: MIX (Mixed Subpopulation), NSS (Non-Stiff Stalk), SS (Stiff Stalk), and TST (Tropical/Subtropical). We first evaluated the influence of maize genotype on rhizosphere bacterial and fungal communities by comparing α-diversity indices among the four groups. The results indicated no significant differences in bacterial and fungal richness, Chao1, and Shannon indices among the four maize genotypes (*P* > 0.05, Fig. 1a, Fig. S2). Bacterial β-diversity differed significantly among the four maize genotypes, as revealed by the principal coordinate analysis (PCoA) and the permutational multivariate analysis of variance (PERMANOVA) test (*R* = 0.051, *P* = 0.001, Fig. 1d). Along PCoA1, community similarity in genotype SS and TST was significantly lower than in MIX and NSS (*P* < 0.05, Fig. 1d), while no significant difference was detected between SS and TST or between MIX and TST (*P* > 0.05, Fig. 1d). Along PCoA2, genotype SS showed higher community similarity than TST, NSS, and MIX (*P* < 0.05, Fig. 1d), while no significant difference was observed between TST and NSS (*P* > 0.05, Fig. 1d). In contrast, no significant difference in fungal community β diversity was observed among the four genotypes (*R* = 0.006, *P* = 0.700, Fig. 1e). However, genotype TST showed significantly lower community similarity along PCoA1, and SS showed lower similarity along PCoA2, compared with other genotypes (*P* < 0.05, Fig. 1e). Moreover, bacterial community similarities within SS and TST were significantly higher than those between any two genotypes (*P* < 0.05, Fig. 1b), while similarities within MIX and NSS were significantly lower than between-genotype similarities (*P* < 0.05, Fig. 1b, Table S1), except for the similarity between MIX and NSS, which showed no difference (*P* = 0.900, Fig. 1b, Table S1). For fungi, the within-group similarity of TST was significantly lower than that between any two genotypes (*P* < 0.05, Fig. 1c, Table S1), whereas no consistent pattern was observed for the other genotypes (Fig. 1c, Table S1). These results showed that maize genotypes differentially affect rhizosphere bacterial and fungal community diversity.

Community composition of rhizosphere bacteria and fungi at the phylum level was also examined. The bacterial community was primarily composed of 15 phyla (Fig. S3a), while fungal community was mainly composed of 12 phyla (Fig. S3b). Clustering analysis of bacterial and fungal phyla showed that the phylum composition of genotype SS differed the most from that of the other three genotypes (Fig. S3). Differential analyses demonstrated statistically significant differences in the relative abundances of both rhizosphere bacterial and fungal genera among the four maize genotypes (*P* < 0.05, Fig. S4).

### Rhizosphere bacterial and fungal community assembly among maize genotypes

**Fig. 2:**
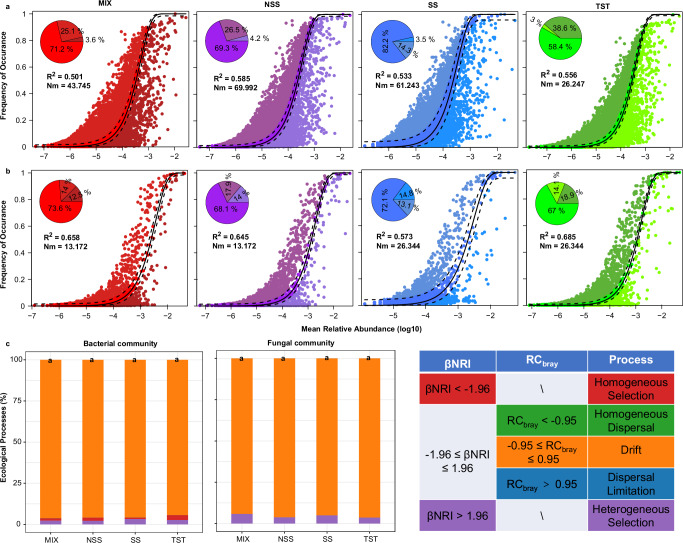
Assembly of rhizosphere bacterial and fungal communities among four maize genotypes. Neutral community models (NCMs) of rhizosphere (**a**) bacterial and (**b**) fungal community assembly among four genotype maize plants. The solid black lines indicate the best fit of the models, and the dashed black lines represent 95% confidence intervals. *R*^2^ indicates the fit of the model, and Nm indicates the metacommunity size times immigration. **c** Relative importance and percentage change of bacterial and fungal community assembly processes. Both the within-bin beta Net Relatedness Index (βNRI) and the modified Raup-Crick metric (RC) were calculated to estimate the relative contribution. Maize genotypes: MIX, Mixed Subpopulation; NSS, Non-Stiff Stalk; SS, Stiff Stalk; TST, Tropical/Subtropical.

Neutral community models (NCMs) were used to assess microbial community assembly across four maize genotypes. Based on neutral theory, the model estimates the relative contribution of stochastic processes to community composition. A higher *R*² value indicates a better fit to the neutral model, suggesting that community assembly is more influenced by stochastic processes and less by deterministic processes. Across all four genotypes, *R*² values for both bacterial and fungal communities range from 0.50 to 0.70 (Fig. 2a, b), indicating that community assembly was governed by a combination of stochastic and deterministic processes, with stochasticity playing the dominant role. Besides, fungal communities exhibited stronger stochastic assembly patterns than bacterial communities, as reflected by consistently higher *R*² (MIX, *R*² = 0.658; NSS, *R*² = 0.645; SS, *R*² = 0.573; TST, *R*² = 0.685, Fig. 2b). In comparison, *R*² values of bacterial communities were lower (MIX, *R*² = 0.501; NSS, *R*² = 0.585; SS, *R*² = 0.533; TST, *R*² = 0.556, Fig. 2a).

To further elucidate the mechanisms underlying the stochastic assembly of bacterial and fungal communities, we evaluated the relative contributions of five ecological processes using the Infer Community Assembly Mechanisms by Phylogenetic-bin-based null model (iCAMP) framework (Fig. 2c). The results showed that drift, a stochastic process associated with random demographic fluctuations, dominated the assembly of both bacterial and fungal communities, accounting for over 90% of the total contribution (Fig. 2c). Moreover, no significant differences were observed in the relative importance of ecological processes among the four maize genotypes for either bacterial or fungal communities. These findings are consistent with the results from the NCMs, reinforcing the results that rhizosphere microbial community assembly is primarily driven by stochastic processes.

### Network analysis for selecting core strains of rhizosphere bacterial and fungal communities among maize genotypes

**Fig. 3:**
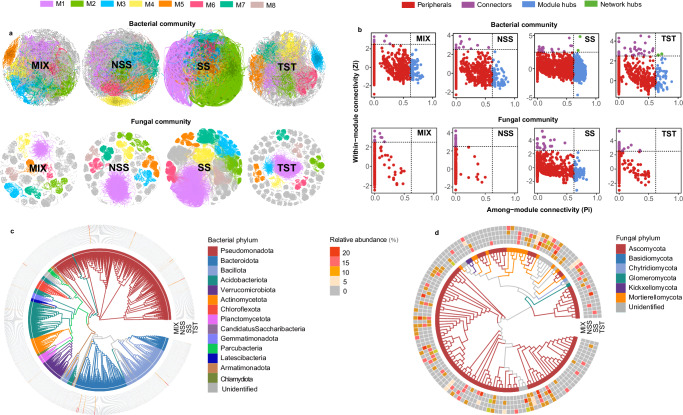
Network analysis of rhizosphere bacterial and fungal communities among four maize genotypes. **a** Network graphs of rhizosphere bacterial and fungal communities among four genotype maize plants. Different colors represent different network modules. **b** Zi-Pi plot of bacterial and fungal communities among four genotype maize plants. Phylogenetic tree of keystone species in (**c**) bacterial and (**d**) fungal communities. Maize genotypes: MIX Mixed Subpopulation, NSS Non-Stiff Stalk, SS Stiff Stalk, TST Tropical/Subtropical.

Microbial network analysis further revealed differences in microbial communities among four maize genotypes (Fig.3a). Network topological properties of bacterial and fungal communities, such as node numbers, edges numbers, diameter, average degree and module numbers, were different among four maize genotypes (Table S2). Notably, the networks of both bacterial and fungal communities of genotype SS exhibited distinct differences compared to other genotypes (Fig. 3a). The rhizosphere bacterial networks were more complex than the fungal networks across maize genotypes, as indicated by higher numbers of nodes, edges, and keystone taxa (Fig. 3a, Table S2). Based on network topological properties, the Zi-Pi method was used to find the key species that maintain the structure and function of microbial communities. The results showed that Zi-Pi characteristics of bacterial and fungal communities were also different among four maize genotypes (Fig. 3b, Table S3). Phylogenetic analysis and species annotation were conducted on the key nodes of bacterial and fungal community networks to identify these critical species. Specially, the results showed that the key nodes in bacterial community networks primarily consisted of 14 phyla (Fig. 3c), and the key nodes in fungal community networks were mainly composed of 6 phyla (Fig. 3d).

**Fig. 4:**
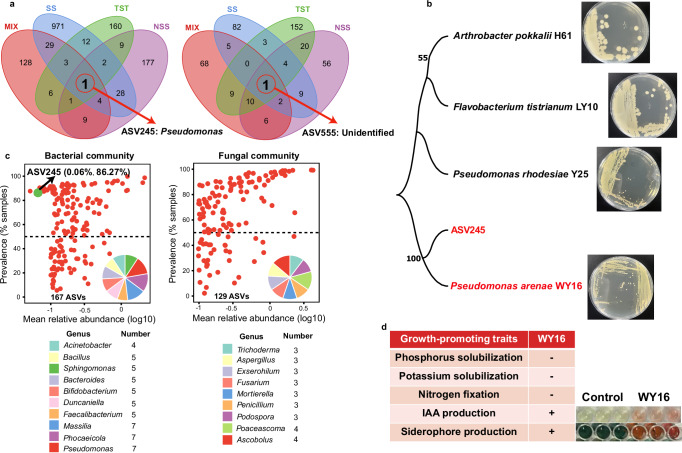
Identifying core rhizosphere bacterial and fungal microbiomes among four maize genotypes. **a** Shared keystone species in rhizosphere bacterial and fungal communities among four genotypes. **b** The phylogenetic tree of ASV245 and four strains (WY16, H61, LY10 and Y25). **c** Abundance-prevalence distribution of the bacterial ASV set and the fungal ASV set. The pie chart depicts bacterial genera occurring ≥ 3 times and fungal genera occurring ≥ 2 times, with different colors represent different genera. **d** Potential growth-promoting traits of WY16. Maize genotypes: MIX Mixed Subpopulation, NSS Non-Stiff Stalk, SS Stiff Stalk, TST Tropical/Subtropical.

Based on the results of key nodes in bacterial and fungal community networks, Venn plots showed that ASV245 was consistently present across all four maize genotypes in the bacterial communities, while an unidentified ASV 555 was observed in the fungal communities of these genotypes (Fig. 4a). This finding highlights the widespread occurrence of ASV245 across bacterial communities associated with different maize genotypes. Taxonomic annotation and BLAST analysis identified ASV245 as belonging to the genus *Pseudomonas* (Fig. S5a). Among bacterial genera, *Pseudomonas* exhibited one of the highest relative abundances across all four maize genotypes (Fig. S6), suggesting a key role in shaping the rhizosphere microbiome. Based on prevalence (≥ 85%) or mean relative abundance (≥ 0.1%) thresholds, seven *Pseudomonas* ASVs were identified, representing one of the largest proportions among the 167 core ASVs (Fig. 4c). Notably, ASV245 displayed a mean relative abundance of 0.06% and a prevalence of 86.27% across all samples (Fig. 4c), consistent with the network analysis.

### Screening of core strains and their growth-promoting effects on maize

**Fig. 5:**
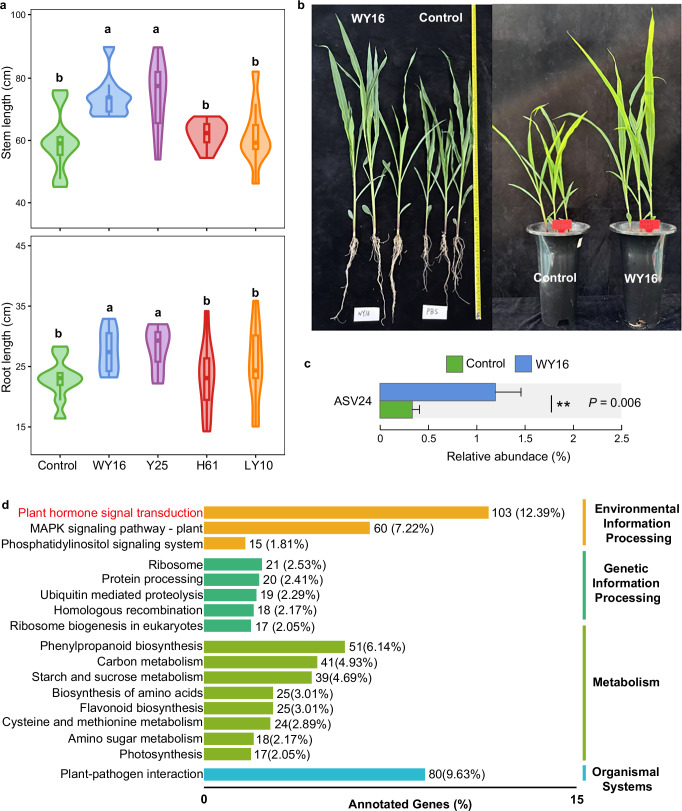
Functional validation and analysis of WY16 growth-promoting effects. **a** Effect of inoculating different strains on corn stem and root length in pot experiments. **b** Comparison of maize inoculated with WY16 and the control group in pot experiments. **c** Relative abundance of ASV24 in the WY16-inoculated group and the control group in the pot experiment. **d** KEGG enrichment analysis of differentially expressed genes. The vertical axis on the left represents the names of the KEGG metabolic pathways, and the horizontal axis indicates the number of genes annotated to that pathway and the proportion of that number relative to the total number of annotated genes.

To isolate strains corresponding to ASV245, selective media targeting for *Pseudomonas* were employed. Six *Pseudomonas* strains (WY16, Y25, F4, B5, H59, and H62) were obtained from maize rhizosphere soil (Table S4). Phylogenetic analysis revealed that ASV245 shares the closest evolutionary relationship with strain WY16 (Figs. 4b, S5j). Further sequence alignment between *P. arenae* WY16 and ASV245 showed only 20 nucleotide mismatches out of 427 base pairs (Fig. S7a), indicating that WY16 was highly similar to ASV245. Therefore, WY16 and Y25 (*P. rhodesiae*) were selected as representative *Pseudomonas* isolates for plant growth-promoting assays. In addition, two phylogenetically distant strains, *Arthrobacter pokkalii* H61 and *Burkholderia paludis* LY10, were included as non-*Pseudomonas* controls to determine whether the growth-promoting effects were genus-specific. Characterization of the potential growth-promoting traits of WY16 revealed that it can produce IAA and siderophores (Fig. 4d). Functional validation of the four strains for their effects on maize growth (Fig. 5a). The results showed that WY16 and Y25 significantly promoted maize stem and root growth compared with the control (*P* < 0.05, Fig. 5a), whereas H61 and LY10 exhibited no significant effect (*P* > 0.05, Fig. 5a). Consistently, field experiments confirmed that WY16 significantly enhanced maize stem height (*P* = 0.017, Table S5).

Analysis of the rhizosphere bacterial communities from maize treated with WY16 and the control group in pot experiment revealed that WY16 shared a close phylogenetic relationship of 94.17% with *Pseudomonas*-associated ASV24 (Figs. S7b, S8). Further comparison of ASV24 relative abundance showed that it was significantly higher in the WY16-inoculated group than in the control (Fig. 5c), suggesting strain WY16 successfully colonized and was enriched in the maize rhizosphere. To further confirm the promoting effect of WY16 on maize, RNA-seq analysis was performed on maize roots from both the WY16-inoculated and control groups. Principal component analysis (PCA) of gene expression profiles demonstrated clear separation between the two treatments, with high within-group similarity and distinct intergroup differences (Fig. S9a). Differential expression analysis identified 1523 significantly upregulated and 1419 significantly downregulated genes in the WY16 group compared to the control (Fig. S9b). These differentially expressed genes were mapped to the Kyoto Encyclopedia of Genes and Genomes (KEGG) database to identify enriched metabolic pathways. Seventeen significantly enriched pathways with annotation rates greater than 2% were identified (Fig. 5d). Notably, the most significantly enriched pathway was plant hormone signal transduction. Further analysis of this pathway revealed 23 genes were significantly upregulated and 19 genes were downregulated in the WY16-treated group compared to the control (Fig. S10). Inoculation with WY16 activated a complex network of phytohormone signaling pathways, including those involved in auxin, cytokinin, gibberellin, ethylene, jasmonic acid, and salicylic acid responses. Within the auxin signaling pathway, genes encoding AUX1 (Auxin Influx Carrier), TIR1 (Transport Inhibitor Response 1), and ARF1 (Auxin Response Factor 1) were significantly upregulated. Additionally, the AUX/IAA, GH3 (Gretchen Hagen), and SAUR (Small Auxin-Up RNA) gene families also showed strong upregulation (Fig. S10).

## Discussion

Maize genotype plays a crucial role in shaping the composition and function of the rhizosphere microbial community. This influence is regulated not only by environmental factors such as soil fertility, but also by plant genetic characteristics^6,22^. However, most previous studies have primarily focused on a limited number of maize genotypes. For instance, previous research investigated how maize genotypes carrying the *su1* and *sh2* alleles affect rhizosphere microbial structure, biomass, activity, and growth^22^. Correlations among genetic relatedness, plant phenotypic traits, and the composition of soil bacterial and fungal communities using six maize cultivars developed over the past six decades were explored^6^. Despite these valuable insights, large-scale studies on rhizosphere microbial communities across diverse maize genotypes remain limited.

To comprehensively explain how maize genotype influences the characteristics of the rhizosphere microbial community, we analyzed 1005 rhizosphere microbial communities derived from 335 genetically distinct maize populations across four genotype groups. Our results demonstrate that maize genotypes exhibit distinct differences in rhizosphere bacterial and fungal community diversity and composition. Specially, maize genotype had no significant effect on bacterial and fungal α-diversity but it significantly influenced bacterial β-diversity, while fungal β-diversity remained largely unaffected. Bacterial communities of SS and TST genotypes were more internally homogeneous, while fungal community differences among maize genotypes were relatively weak and inconsistent (Fig. 1). These results suggest that long-term co-evolution between maize and its associated microbiome has shaped the rhizosphere bacterial and fungal communities, resulting in diverse rhizosphere microbial communities across different maize genotypes. Previous studies have also demonstrated that under different nitrogen treatment levels, the response of the rhizosphere microbial community to nitrogen input differs between maize inbred lines and hybrids. High nitrogen input increases microbial diversity in the rhizosphere of inbred maize lines but decreases microbial diversity in hybrid maize^23^. Besides, plant genotypes can influence the structure and function of microbial communities by altering the chemical and physical properties of the rhizosphere environment^24^. Given that our samples were collected from the same region and under the same pedological conditions, the observed differences in rhizosphere microbiota are likely attributable primarily to maize genotypic variation. Further taxonomic analysis revealed significant genotype-specific differences in both bacterial and fungal community composition at the phylum level, particularly in genotype SS compared to the other three genotypes (Fig. S3). At the genus level, the relative abundance of several bacterial and fungal genera also varied markedly among genotypes (Fig. S4), highlighting the selective influence of host genotype on microbial recruitment. These findings are consistent with previous studies demonstrating that plant genotype can further alters the abundance of specific microbial taxa in the rhizosphere^25^.

Although the rhizosphere microbial communities differed among maize genotypes (Fig.1), the assembly processes of both bacterial and fungal communities were predominantly governed by stochastic processes (Fig. 2). Neutral theory posits that all species are ecologically equivalent and that community structure is shaped by stochastic processes such as birth, death, dispersal, extinction, and speciation events. In this context, stochasticity reflects a balance between microbial loss and gain, driven by chance rather than selection^13,26^. In contrast, niche theory emphasizes deterministic processes, which involve both biotic and abiotic factors. These include species interactions such as competition, predation, and mutualism, as well as environmental filtering imposed by variables like salinity, pH, and temperature^27,28^. Our findings demonstrate a dominance of stochastic processes in rhizosphere community assembly regardless of maize genotype (Fig. 2c). This suggests that stochastic forces may outweigh host-driven selective pressures in shaping rhizosphere microbial communities. Together, despite significant genotype-dependent variation in microbial diversity and composition, both NCMs and iCAMP-based ecological analyses revealed that community assembly was predominantly governed by stochastic processes. This indicates a high degree of assembly robustness and evolutionary conservation in rhizosphere microbiota, largely independent of host genetic background. These results highlight the ecological stability of rhizosphere microbial communities and support the potential existence of genotype-independent core microbes.

In the different genotypes plant rhizosphere, although the microbial communities are highly diverse, certain key microbial taxa consistently persist and are referred to as the core microbes^21^. These microbes often fulfill essential ecological functions such as nitrogen fixation, phosphorus solubilization, disease suppression, and plant growth promotion^18^. Previous studies have showed that distinct plant genotypes can stably maintain a subset of core microbiota under various soil conditions. For example, PGPR such as *Pseudomonas spp*., *Bacillus spp*., and *Arthrobacter spp*. are consistently enriched in the rhizosphere across multiple maize genotypes, suggesting their close association with maize development^6^. Through Zi-Pi network analysis^29^, we identified keystone microbial species inhabiting the rhizosphere of different maize genotypes (Fig. 3). Subsequent Venn diagram analysis revealed one shared ASV245 that consistently present across all genotypes (Fig. 4a). According to the prevalence-abundance framework, ASV245 exhibited both high prevalence (86.27%) and high mean relative abundance (0.06%) (Fig. 4c). Taxonomic annotation assigned ASV245 to the genus *Pseudomonas*. Consistently, Genus-level analysis showed that *Pseudomonas* displayed the highest relative abundance among four maize genotypes (Fig. S6a). Members of *Pseudomonas* are widely recognized as plant-beneficial microbes^30^. For instance, *Pseudomonas* TR47 has been reported to alleviate drought stress in plants by modulating the rhizosphere microbial community^31^. *Pseudomonas* are capable of producing a variety of secondary metabolites, such as hydrogen cyanide and phenazine compounds, which suppress phytopathogens and enhance plant disease resistance^32^.

To further verify the functional role of *Pseudomonas* in promoting maize growth, we performed both pot and field experiments to evaluate the plant growth-promoting effects of strain WY16. In both experiments, WY16 significantly enhanced maize growth compared with the control group (Fig. 5a, b, Table S5), demonstrating its beneficial effect on maize growth. These results are consistent with previous findings that fluorescent *Pseudomonas* RRF3 can modulate maize root structure through root exudate-mediated interactions^33^. Interestingly, phylogenetic tree construction and sequence alignment revealed a high sequence similarity (94.17%) between one of our isolates (WY16) and ASV24 (Figs. S7b, S8). High-throughput bacterial sequencing data revealed that strain WY16 effectively colonized the maize rhizosphere (Fig. 5c). Transcriptomic analysis further demonstrated that, compared to the control, WY16 inoculation significantly enriched metabolic pathways associated with plant hormone signal transduction, particularly those involved in auxin biosynthesis (Figs. 5d, S10). Collectively, these findings indicate that WY16 can colonize the maize rhizosphere and promote plant growth by modulating maize hormone signaling pathway. Previous studies have shown that PGPR can influence plant development by modulating phytohormone signaling pathways^34^. Among these, auxins, especially IAA, play a pivotal role throughout the plant life cycle by regulating cell division, elongation, differentiation, and root development^35,36^. Our results also showed that WY16 can produce IAA (Fig. 4d). Despite significant differences in rhizosphere microbial communities across maize genotypes, certain microbial taxa, such as *Pseudomonas*, consistently persists as core microbiome by promoting maize growth. Although *Pseudomonas* species have been widely reported as PGPR in maize and other crops^33,34^, our study provides new conceptual insight into the ecological mechanisms underlying their persistence. Specifically, we demonstrate that *Pseudomonas* functions as a genotype-independent core taxon across diverse maize genotypes and link this stability to stochastic community assembly processes. This finding suggests that the maintenance of conserved, functionally important microbial taxa may not only depend on host genotype selection but also by stochastic ecological processes. Together, our results indicate that the persistence of *Pseudomonas* as a core taxon across diverse maize genotypes may be influenced not only by host-associated selection but also by stochastic community assembly processes. Although all maize genotypes were cultivated in the same soil to minimize environmental variation, we cannot exclude the possibility that the conserved presence of *Pseudomonas* reflects adaptation to specific soil physicochemical properties rather than complete genotype independence. Future experiments growing the same maize genotypes across soils with contrasting properties and environmental conditions will be crucial to disentangle the relative contributions of soil and host factors.

**Fig. 6:**
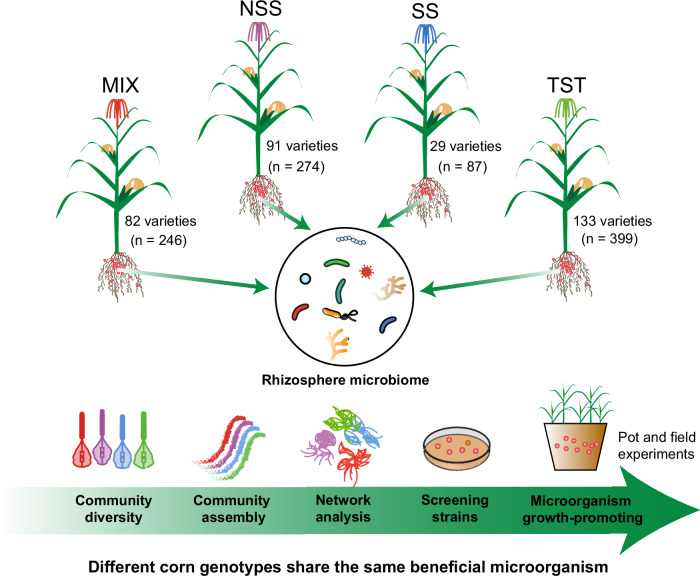
Conceptual diagram of four maize genotypes share the same beneficial microorganism. Maize genotypes: MIX, Mixed Subpopulation; NSS, Non-Stiff Stalk; SS, Stiff Stalk; TST, Tropical/Subtropical.

Taken together, this study demonstrates that although host genotype significantly influences the diversity and composition of rhizosphere microbial communities, their assembly is largely governed by stochastic processes. This evolutionary robustness indicates that rhizosphere microbiomes maintain a conserved assembly framework despite host genetic variation. Through network analysis and prevalence-abundance framework, we identified core microbial members and isolated *Pseudomonas* strain WY16, which exhibited a consistent growth-promoting effect on maize (Fig. 6). These results highlight the potential of *Pseudomonas* WY16 as a genotype-independent bioinoculant. Overall, our findings bridge ecological theory with agricultural application by showing that key microbial taxa can stably associate with genetically diverse hosts and enhance plant performance. This work provides a foundation for improving crop productivity through the targeted use of beneficial microorganisms.

## Methods

### Field experiment design and sample collection

Maize field experimental was conducted at Yazhou District of the Beijing Academy of Agriculture and Forestry Sciences, Hainan, China (109.159°E, 18.39°N). The soil had a pH of 6.5 ± 0.2, total potassium of 3.58 ± 0.09%, total phosphorus of 0.15 ± 0.01%, total nitrogen of 0.82 ± 0.12%, and organic matter content of 1.07 ± 0.23%. Each maize variety was planted in a single row at a density of 100,000 plants/ha, with ten replicates arranged in a randomized block design. Rows were spaced 50 cm apart, with an inter-plant distance of 20 cm. In December 2021, rhizosphere samples were randomly collected from 335 maize varieties representing four genotypes: MIX (Mixed Subpopulation), NSS (Non-Stiff Stalk), SS (Stiff Stalk), and TST (Tropical/Subtropical)^37^. Sampling was conducted at the V10 growth stage, yielding a total of 1,005 samples with three biological replicates per variety. Prior to sampling, the top 3 cm of surface soil was removed to minimize contamination. Whole maize plants were carefully uprooted or excavated using sterile shovels to obtain roots from 6-12 cm depth. The maize roots were immediately placed on ice and transported to the laboratory. Maize roots were gently shaken to remove loosely attached soil, followed by meticulous brushing to eliminate tightly bound soil particles. Approximately 4 g of root segments (4.5–10.5 cm in length) were then excised and placed in 50 mL sterile centrifuge tubes containing 40 mL PBS. Samples were shaken at 180 rpm for 30 min and vortexed for 30 s to dislodge microbes. After centrifugation at 10,000 g for 10 min, the supernatant was discarded and the microbial pellet was stored at -80°C for subsequent analysis.

### DNA extraction and amplification sequencing

DNA was extracted using the MoBio Power Soil Extraction Kit (MoBio Laboratories, Carlsbad, CA, USA). The extraction procedure was performed according to the manufacturer’s instructions. The quality and concentration of the extracted DNA were assessed using 1% agarose gel electrophoresis and a NanoDrop One UV-Vis spectrophotometer (Thermo Fisher Scientific, Wilmington, USA) to ensure that the DNA met the required standards. PCR amplification was conducted using the primer pairs 338 F/806 R and ITS1F/ITS2R to target the V3-V4 hypervariable regions of the bacterial 16S rRNA gene and the fungal ITS1 region, respectively. The PCR amplification process followed established protocols as described in previous studies^38^. High-throughput sequencing of the bacterial 16S rRNA and fungal ITS1 regions was performed on the Illumina NovaSeq 6000 platform (Illumina, San Diego, USA).

### Sequencing data processing

QIIME 2 (Quantitative Insights into Microbial Ecology2, Version 2021.8) was used to analyze the raw sequences^39^. The bacterial dataset contained per-sample read counts ranging from 24,727 to 699,756, whereas the fungal dataset ranged from 15,789 to 479,087 reads per sample. Initially, the primers, barcodes, and low-quality sequences of the amplicons were removed using the built-in plugins of QIIME2. Subsequently, the DADA2 (Divisive Amplicon Denoising Algorithm 2) method was employed to remove sequencing noise, erroneous sequences, and chimeric sequences from the amplicon data^40^, resulting in mean read retention rates of 84.10% for bacteria and 76.58% for fungi after quality control and denoising. The high-quality sequences resulting from the DADA2 denoising process were clustered at 100% similarity, ultimately generating Amplicon Sequence Variants (ASVs). Taxonomic annotation of the ASVs for soil bacteria and fungi was performed using the RDP (http://rdp.cme.msu.edu/, version 2.14)^41^ and UNITE (https://unite.ut.ee/, version 10.0)^42^ databases, respectively. Mitochondrial and chloroplast sequences were removed from the ASVs using “qiime taxa filter-table”. Sequence counts were rarefied to a minimum depth of 13,172 for bacteria and 8,749 for fungi (Fig. S1), ensuring sufficient sequencing coverage for downstream community analyses.

### *Pseudomonas* isolation from maize rhizosphere soil

*Pseudomonas* selective medium was employed for the selective isolation of *Pseudomonas* species^43,44^. The medium composition was as follows (g/l): peptone (20), MgCl₂ (1.4), K₂SO₄ (10), Irgasan (0.025), Agar (15), with a pH of 7.0 ± 0.2. This medium selectively inhibits the growth of non-*Pseudomonas* strains by the addition of Irgasan in the medium. Rhizosphere soil from ten maize roots of each genotype was randomly collected and pooled in equal proportions to form one composite sample. The four composite samples were serially diluted in sterile water, and 100 µL aliquots were plated on Tryptic Soy Agar (TSA) and incubated at 28°C for 24–48 h. Distinct bacterial colonies were purified by repeated streaking and preserved in glycerol at −80°C. Isolates were preliminarily characterized by colony and morphology, followed by 16S rRNA gene sequencing and BLAST analysis for taxonomic identification. A total of six non-redundant *Pseudomonas* strains (WY16, Y25, F4, B5, H59, and H62) were obtained from the rhizosphere of the four maize genotypes for subsequent experiments (Table S4).

### Phylogenetic tree construction and sequence alignment

The isolated and purified strains were subjected to 16S rRNA sequencing. The obtained 16S rRNA sequences were analyzed using the BLAST in the NCBI database. The top BLAST hits with the highest sequence similarity scores were selected for further phylogenetic analysis. Phylogenetic tree was conducted using Mega11^45^. Multiple sequence alignment was performed using ClustalW (version 2.1) with default parameters to ensure accurate sequence comparison^46^. The alignment results were subsequently visualized and annotated using ESPript 3.0^47^.

### Maize growth-promoting experiment

Pot experiment was conducted to evaluate the growth-promoting effects of four selected bacterial strains on maize. Strain WY16 was selected for its phylogenetic similarity to the core *Pseudomonas* ASV245, while Y25 was included as a representative *Pseudomonas* strain. Two additional strains, *A. pokkalii* H61 and *B. paludis* LY10, were used as non-*Pseudomonas* controls to assess genus-specific effects on plant growth promotion. Each pot contains 5 kg soil. Maize seeds were surface-sterilized with 5% (v/v) sodium hypochlorite and washed five times with sterile water to ensure complete removal of surface contaminants. The sterilized seeds were sown on plates with MS culture medium^48^. Soil was sterilized via γ-radiation before planting. The concentration of each strain was adjusted to an OD₆₀₀ of 0.01 (10^7^ CFU/ mL), and 1 mL of the strain suspension was diluted in 100 mL water and applied to maize plants. The experimental setup included five treatment groups: four strain treatments and one uninoculated control, with ten biological replicates per treatment. The inoculated and control groups were randomly arranged in a greenhouse maintained at 25 ± 2°C with a photoperiod of 16 h dark/8 h light cycle and relative humidity of 60–70%. After 4 week growth, maize plants were sampled for stem and root length measurements. The rhizosphere soil samples for bacterial community analysis and maize root samples for transcriptome were collected. The bacterial community DNA extraction, amplification, and sequencing data processing were performed using the methods described above.

Field experiment was conducted to evaluate the growth-promoting effects of strain WY16 on maize at the Fangshan Research Station of the Beijing Academy of Agriculture and Forestry Sciences (Beijing, China; 39°42′09.1″N, 116°00′46.8″E). Two treatments (WY16 inoculation and non-inoculated control) were established. WY16 was applied on the 7th and 14th days after maize germination, while control received sterile PBS. The inoculum concentration was consistent with that used in the pot experiment. Each plot (2 m × 3 m) contained 20 maize seedlings transplanted at equal spacing. Border rows were used as guards, and maize from these border rows were excluded from sampling. The experiment was arranged in a randomized complete block design with two treatments. Each treatment was replicated three times, resulting in a total of six experimental plots. Field management followed local conventional tillage practices. The experiment was conducted from May 20 to September 20. At harvest, ten plants per treatment were randomly selected to measure stem height, plant weight, stem diameter, ear height, and ear weight.

### Transcriptome sequencing and analysis

Three maize roots per treatment were randomly selected and rinsed with sterile PBS. After blotting residual moisture, the samples were carefully transferred into sterile cryovials, flash-frozen in liquid nitrogen for approximately 30 minutes, and then stored at −80 °C for RNA extraction. Total RNA was extracted using the Tiangen RNAprep Pure Plant Kit (Tiangen, Beijing, China) according the instructions provided by the manufacturer and assessed for quality following the protocol described by previous research^49^. Each treatment included three biological replicates, and RNA sequencing was performed on the Illumina NovaSeq 6000 platform with a 150 bp paired-end (PE150) configuration. Raw sequencing reads were processed using “fastp” (v0.19.7) to remove adapter sequences from the 3′ ends and to discard reads with an average quality score below Q20^50^. The maize reference genome sequence and corresponding gene annotation files were downloaded for downstream analysis. “HISAT2” (v2.1.0) was used to build the genome index and align the high-quality clean reads to the reference genome^51^. Gene-level quantification was performed using “HTSeq” (v 2.0) to calculate the read counts for each gene^52^. Gene expression levels were quantified using FPKM (fragments per kilobase of transcript per million mapped reads) method.

### Statistical analyses

All analyses were conducted in R (version 4.4.1). Richness, Chao1, and Shannon indices of soil bacterial and fungal communities were calculated using the “estimate” and “diversity” functions in the vegan package after removing ASVs with relative abundances below 0.005%^53^. Subsequently, Bray-Curtis distance matrices were computed using the “vegdist” function in vegan. Within- and between-group community similarities were then calculated following previously described methods^54^. Principal Coordinates Analysis (PCoA) was then performed to assess the β-diversity of soil bacterial and fungal communities. Finally, Permutational Multivariate Analysis of Variance (PERMANOVA) was conducted using the “adonis” function in the vegan package to test for significant differences in β-diversity among groups. One-way ANOVA and Tukey’s HSD tests were performed to assess the differences in α-diversity.

Neutral community models (NCMs) were employed to assess the potential importance of stochastic processes on bacterial and fungal community assembly^55,56^. This model exhibits the relationship between the relative abundance of ASVs and their occurrence frequency. The optimal fitting values of NCMs are displayed within the dashed lines in the figure. The dashed lines represent the 95% confidence interval of the model, which was estimated using bootstrapping with 999 replicates.

Infer Community Assembly Mechanisms by Phylogenetic-bin-based null model (iCAMP) was used to quantify the ecological processes^57,58^. Both the within-bin beta Net Relatedness Index (βNRI) and the modified Raup-Crick metric (RC) were calculated to estimate the relative contribution of homogeneous selection (HoS, βNRI < − 1.96), heterogeneous selection (HeS, βNRI > 1.96), homogeneous dispersal (HD, RC < −0.95 and |βNRI | ≥ −1.96), drift (DR, −0.95 ≤ |RC | ≤ 0.95), and dispersal limitation (DL, RC > 0.95 and |βNRI | ≤ 1.96) in the microbial community assembly.

Spearman correlation coefficients between ASVs were calculated using the “rcorr” function from the Hmisc package. The resulting correlation matrix was adjusted for multiple testing using the “p.adjust” function, with the False Discovery Rate (FDR) method applied for *P*-value correction. Only Spearman correlation coefficients with |*R* | > 0.6 and *P* < 0.05 after correction were retained. Gephi platform was used for network visualization^59^. The topology of each node (ASV) could be measured by its within module connectivity (Zi = 2.5) and among module connectivity (Pi = 0.62). According to the simplified categorization, all nodes in the networks were distributed among four subcategories: Peripherals, Connectors, Module hubs and Network hubs^29^. Nodes that belonged to Connectors, Module hubs and Network hubs were considered to be keystone microbiomes in the network^60^.

The core rhizosphere microbiome across the four maize genotypes was identified using an prevalence-abundance approach^61,62^. ASVs were retained if they were present in at least 85% of all samples or had a minimum mean relative abundance of 0.1% across all samples.

## Supplementary information

Supplementary Material

## Data Availability

Bacterial 16S rRNA and fungal ITS1 sequences are available at ScienceDB (10.57760/sciencedb.29129). Additional data and code are available on GitHub (https://github.com/fxtranquility/R-code).
