# Supplementary material for: Conserved genotype-independent rhizobacteria promote maize growth
Source: NPJ Biofilms Microbiomes. 2025 Dec 31;12:29. doi: 10.1038/s41522-025-00895-4 (PMC12855845; doi:10.1038/s41522-025-00895-4)

Table S1 Comparison of bacterial and fungal community similarity within and between four maize genotypes

| Group | Within-groups1 | Within-groups2 | Between-groups | *P* value |
| --- | --- | --- | --- | --- |
| Bacterial  Community  Similarity | MIX: 0.2100a | NSS: 0.2027a | MIX vs NSS: 0.2062a | 0.900 |
|  | MIX: 0.2100c | SS: 0.2251a | MIX vs SS: 0.2109b | < 0.001 |
|  | MIX: 0.2100c | TST: 0.2597a | MIX vs TST: 0.2290b | < 0.001 |
|  | NSS: 0.2027c | SS: 0.2251a | NSS vs SS: 0.2099b | < 0.001 |
|  | NSS: 0.2027c | TST: 0.2597a | NSS vs TST: 0.2253b | < 0.001 |
|  | SS: 0.2251b | TST: 0.2597a | SS vs TST:0.2351b | < 0.001 |
| Fungal  Community  Similarity | MIX: 0.2895c | NSS: 0.2961a | MIX vs NSS: 0.2920b | < 0.001 |
|  | MIX: 0.2895b | SS: 0.3012a | MIX vs SS: 0.2935b | < 0.001 |
|  | MIX: 0.2895a | TST: 0.2630c | MIX vs TST: 0.2737b | < 0.001 |
|  | NSS: 0.2961a | SS: 0.3012a | NSS vs SS: 0.2962a | < 0.001 |
|  | NSS: 0.2961a | TST: 0.2630c | NSS vs TST: 0.2775b | < 0.001 |
|  | SS: 0.3012a | TST: 0.2630c | SS vs TST:0.2763b | < 0.001 |

Table S2 Network topological properties of bacterial and fungal communities among four maize genotypes

| Topological properties | Bacterial community | | | | | Fungal community | | | |
| --- | --- | --- | --- | --- | --- | --- | --- | --- | --- |
|  | MIX | NSS | SS | TST | MIX | | NSS | SS | TST |
| Number of ASVs | 3857 | 4733 | 4434 | 4753 | 2185 | | 3594 | 5547 | 4236 |
| Number of nodes | 1687 | 2246 | 2350 | 2357 | 526 | | 731 | 1335 | 967 |
| Number of edges | 8041 | 12091 | 76453 | 9114 | 2058 | | 6540 | 20519 | 4982 |
| Network diameter | 15 | 14 | 6 | 9 | 8 | | 9 | 21 | 9 |
| Average degree | 9.533 | 10.767 | 65.066 | 7.734 | 7.825 | | 17.893 | 30.74 | 10.304 |
| Graph density | 0.006 | 0.005 | 0.0028 | 0.003 | 0.015 | | 0.025 | 0.023 | 0.011 |
| Clustering coefficient | 0.5 | 0.491 | 0.449 | 0.42 | 0.829 | | 0.863 | 0.689 | 0.883 |
| Average path length | 5.598 | 5.632 | 2.679 | 6.25 | 2.897 | | 2.327 | 3.683 | 3.152 |
| Modularity | 0.781 | 0.778 | 0.579 | 0.745 | 0.927 | | 0.674 | 0.724 | 0.92 |
| Number of modules | 61 | 70 | 14 | 68 | 51 | | 48 | 17 | 62 |

Table S3 Network node types of bacterial and fungal communities among four maize genotypes

| Types of network nodes | Bacterial community | | | | | Fungal community | | | |
| --- | --- | --- | --- | --- | --- | --- | --- | --- | --- |
|  | MIX | NSS | SS | TST | MIX | | NSS | SS | TST |
| Number of peripherals | 1441 | 1943 | 1274 | 2087 | 426 | | 623 | 1225 | 768 |
| Number of connectors | 161 | 181 | 1022 | 106 | 0 | | 0 | 68 | 0 |
| Number of module hubs | 20 | 50 | 26 | 85 | 101 | | 108 | 38 | 199 |
| Number of network hubs | 0 | 0 | 1 | 2 | 0 | | 0 | 0 | 0 |
| Number of keystones | 181 | 231 | 1049 | 193 | 101 | | 108 | 106 | 199 |

Table S4 Strain codes and their taxonomic classification

| Codes | Taxanomy | Sources |
| --- | --- | --- |
| WY16 | *Pseudomonas arenae* | Maize rhizosphere soil |
| Y25 | *Pseudomonas rhodesiae* | Maize rhizosphere soil |
| F4 | *Pseudomonas alvandae* | Maize rhizosphere soil |
| B5 | *Pseudomonas beijingensis* | Maize rhizosphere soil |
| H59 | *Pseudomonas paracarnis* | Maize rhizosphere soil |
| H62 | *Pseudomonas azotoformans* | Maize rhizosphere soil |
| H61 | *Arthrobacter pokkalii* | Laboratory collection |
| LY10 | *Burkholderia paludis* | Laboratory collection |

Table S5 Comparison of maize traits between the control and WY16 treatment groups in the field experiment

| Plant Traits | Control (n = 10) | WY16 (n = 10) | *P* values |
| --- | --- | --- | --- |
| Stem Height (cm) | 299.1 ± 13.62 | 314.7 ± 12.85 | **0.017*** |
| Plant Weight (kg) | 0.81 ± 0.11 | 0.86 ± 0.10 | 0.318 |
| Stem Diameter (cm) | 2.32 ± 0.16 | 2.43 ± 0.21 | 0.211 |
| Ear Height (cm) | 160.57 ± 12.36 | 161.62 ± 7.68 | 0.822 |
| Ear Weight (kg) | 0.255 ± 0.04 | 0.3 ± 0.07 | 0.091 |

Note: Bold numbers indicate a statistically significant difference. Weight refers to fresh weight.


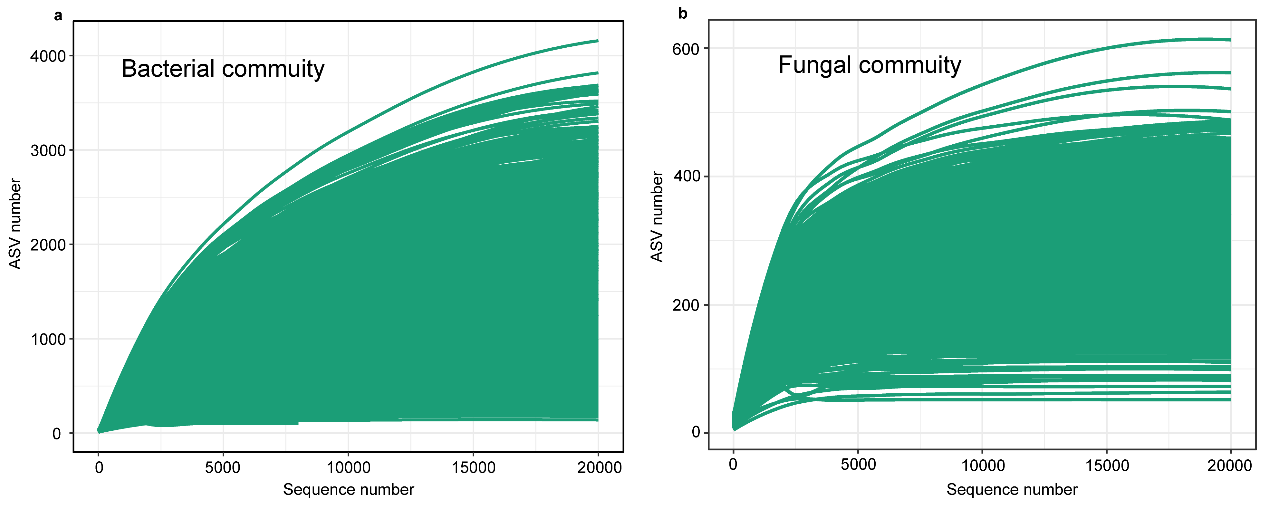


Fig. S1 Rarefaction curve for (a) bacterial and (b) fungal community.


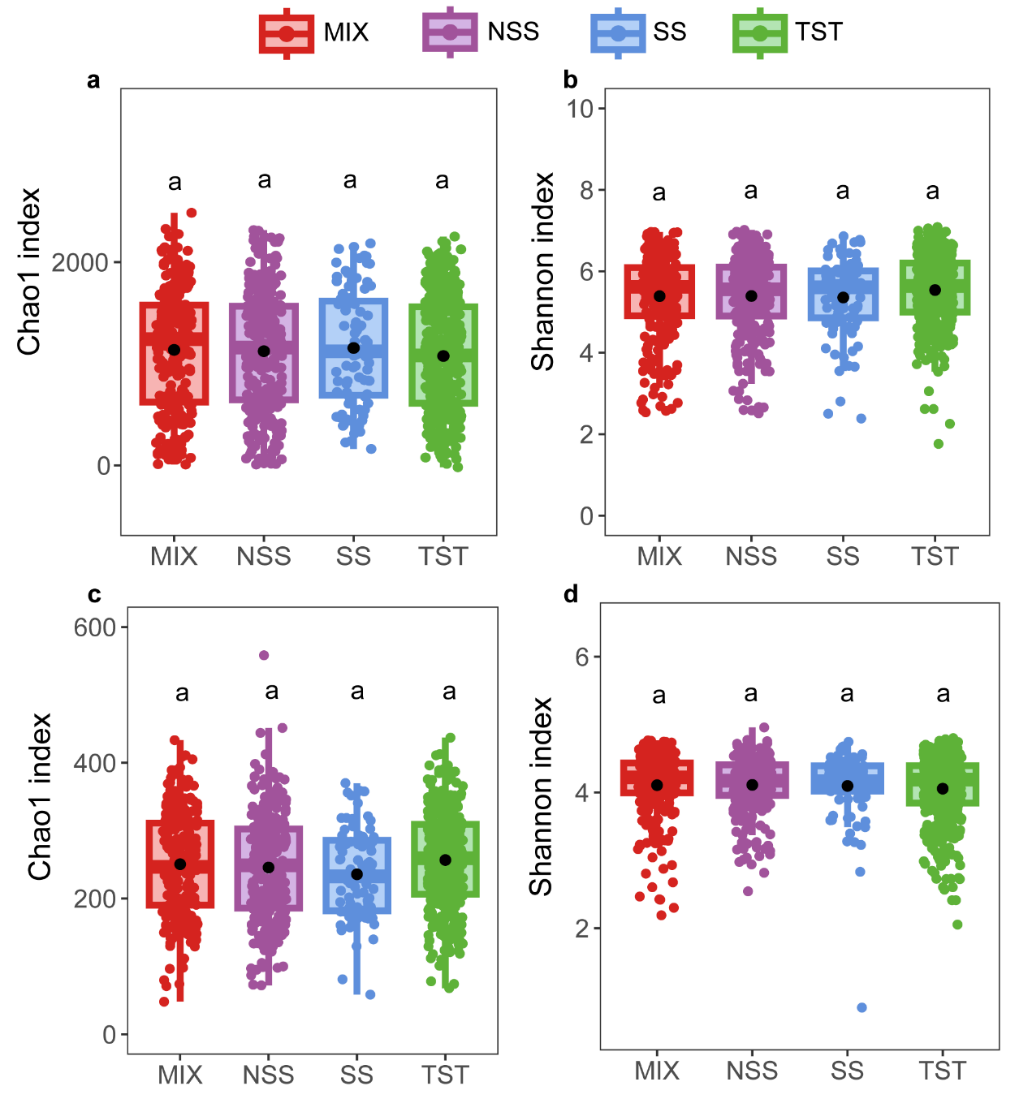


Fig. S2 Chao1 and Shannon indices for (a, b) bacterial and (c, d) fungal communities across four maize genotypes. Lowercase letters indicate statistical differences (*P* < 0.05). Maize genotypes: MIX, Mixed Subpopulation; NSS, Non-Stiff Stalk; SS, Stiff Stalk; TST, Tropical/Subtropical.


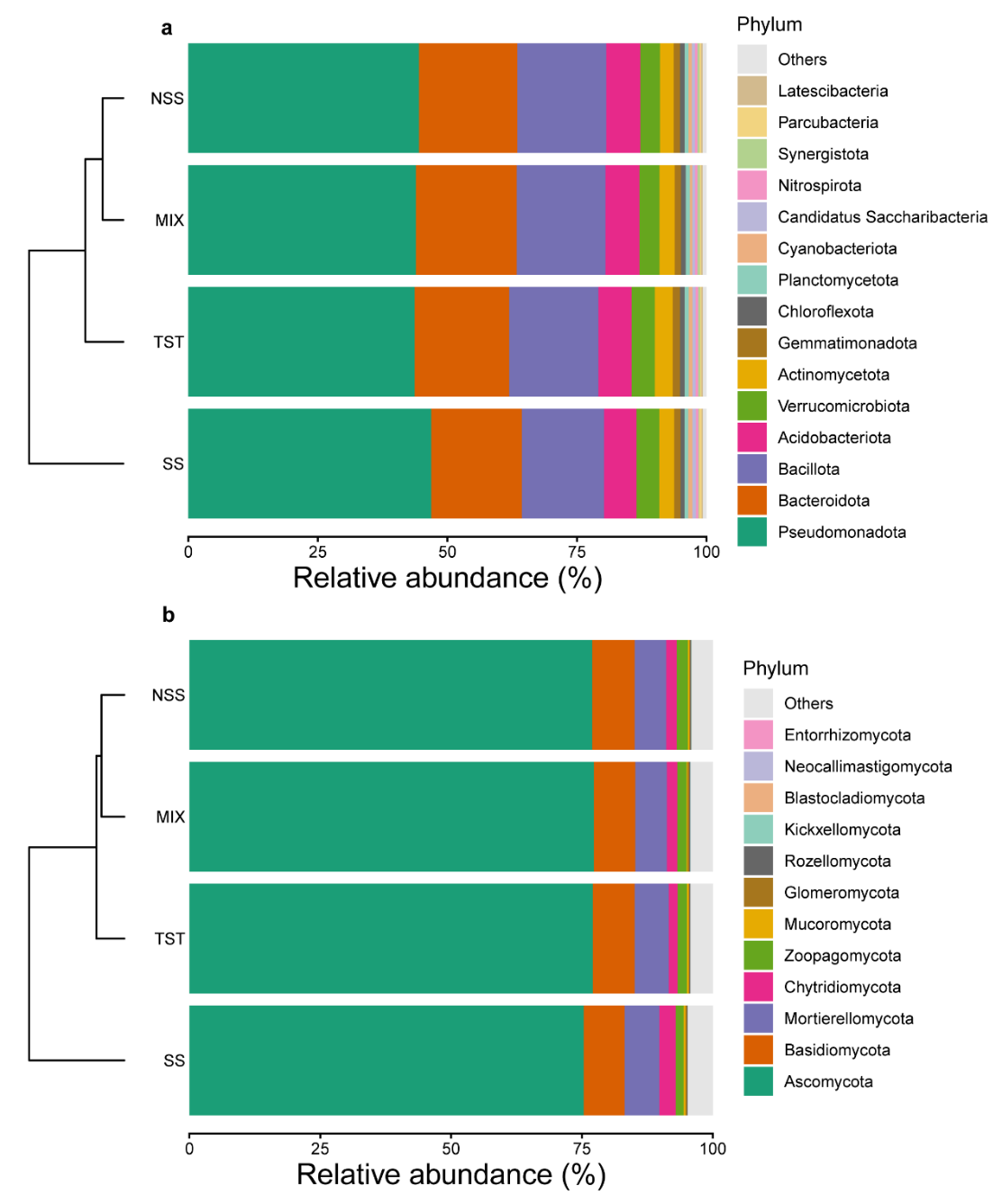


Fig. S3 The phylum composition of (a) bacterial and (b) fungal communities among four genotype maize plants. Different colors within the bar charts represent different taxonomic groups. Maize genotypes: MIX, Mixed Subpopulation; NSS, Non-Stiff Stalk; SS, Stiff Stalk; TST, Tropical/Subtropical.


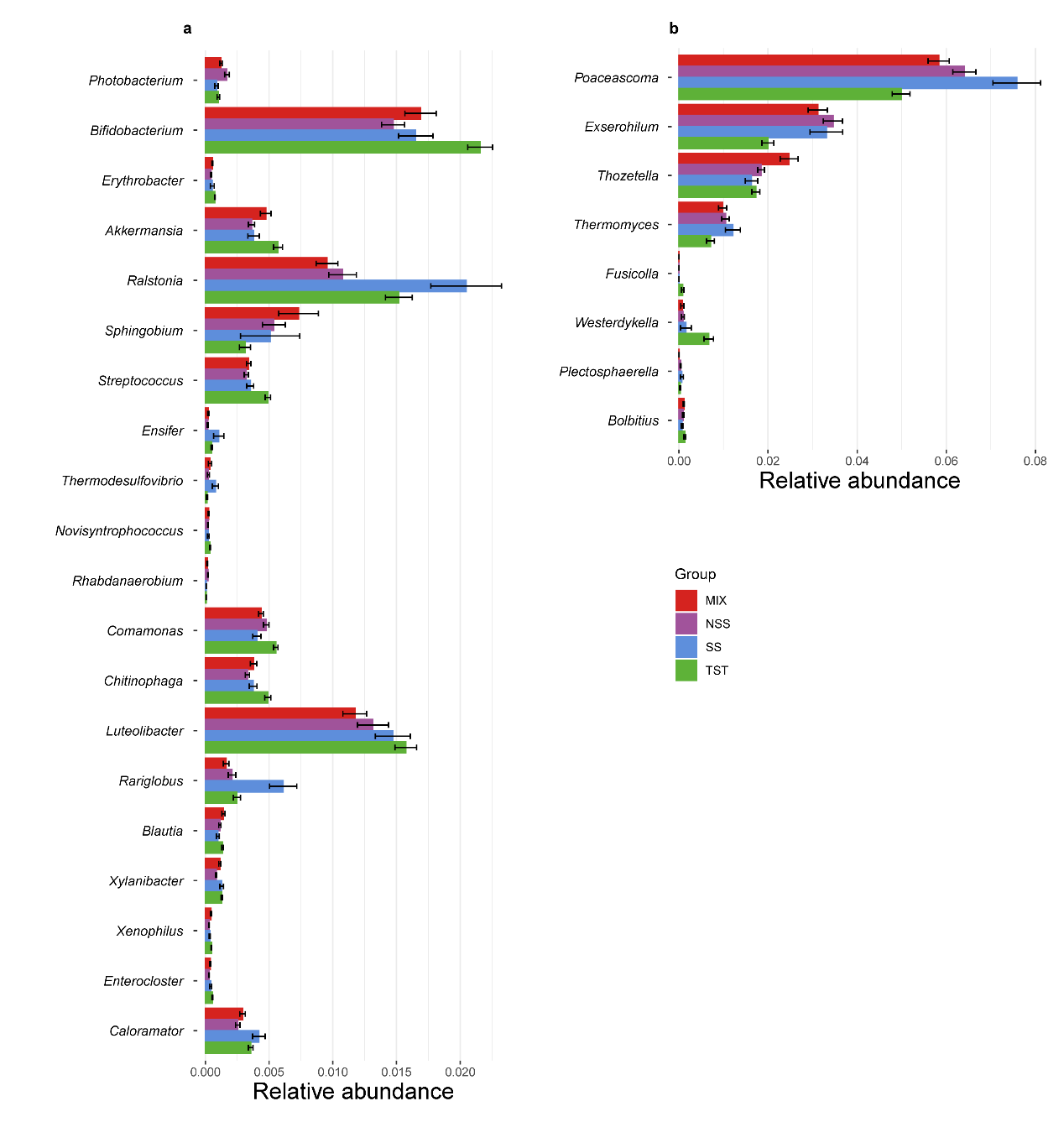


Fig. S4 Distinct rhizosphere (a) bacterial and (b) fungal genera among four genotype maize plants. Maize genotypes: MIX, Mixed Subpopulation; NSS, Non-Stiff Stalk; SS, Stiff Stalk; TST, Tropical/Subtropical.


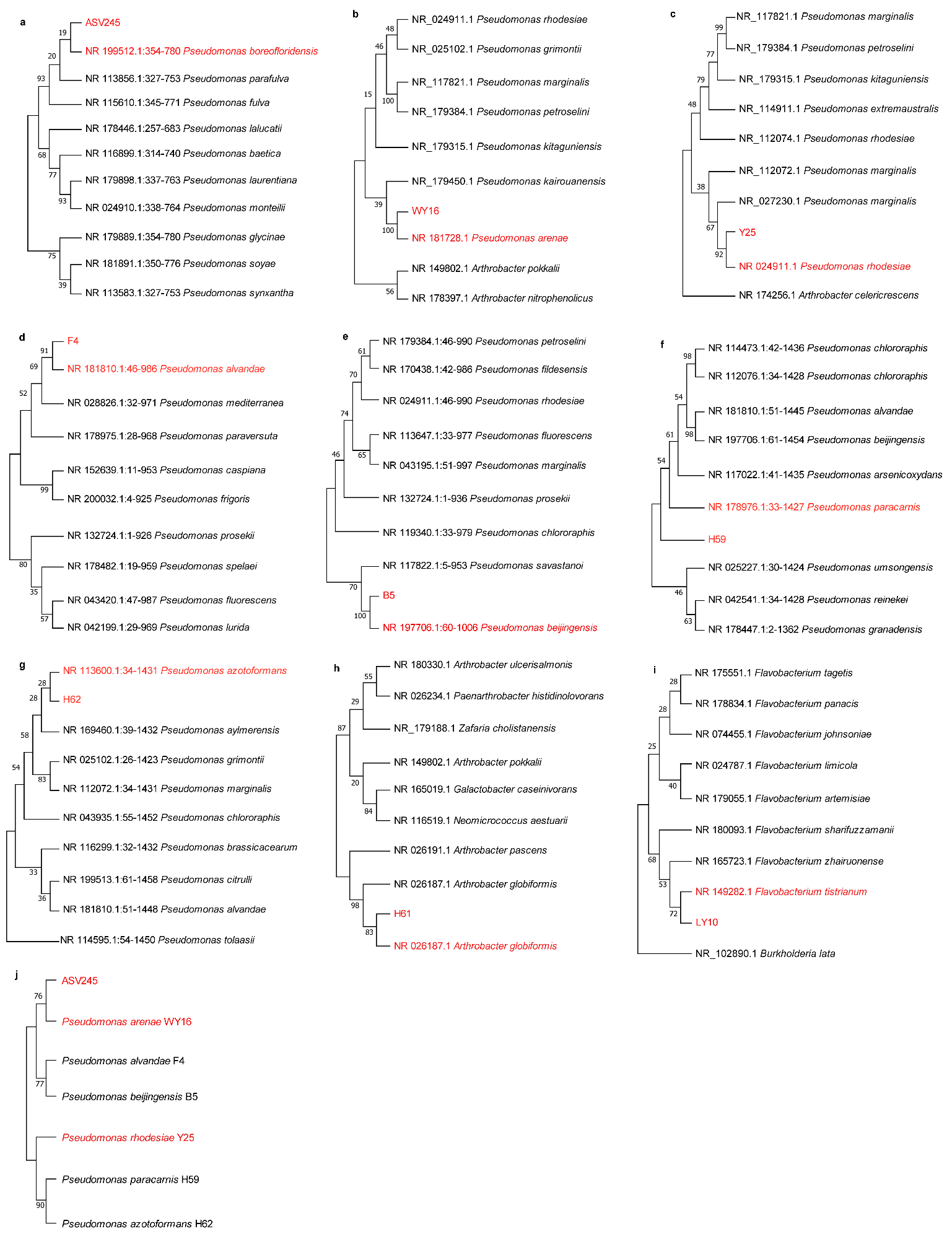


Fig. S5 Phylogenetic trees. (a) ASV245 was most closely related to *Pseudomonas boreofloridensis*. (b) WY16 was most closely related to *Pseudomonas arenae.* (c) Y25 showed the closest similarity to *Pseudomonas rhodesiae*. (d) F4 was most closely related to *Pseudomonas alvandae*. (e) B5 was most closely related to *Pseudomonas beijingensis*. (f) H59 was most closely related to *Pseudomonas paracarnis.* (g) H62 was most closely related to *Pseudomonas azotoformans*. (h) H61 showed the closest similarity to *Arthrobacter pokkalii*. (i) LY10 demonstrated the closest phylogenetic relationship to *Flavobacterium tistrianum*. (j) Phylogenetic relationship between ASV245 and isolated *Pseudomonas* strains.


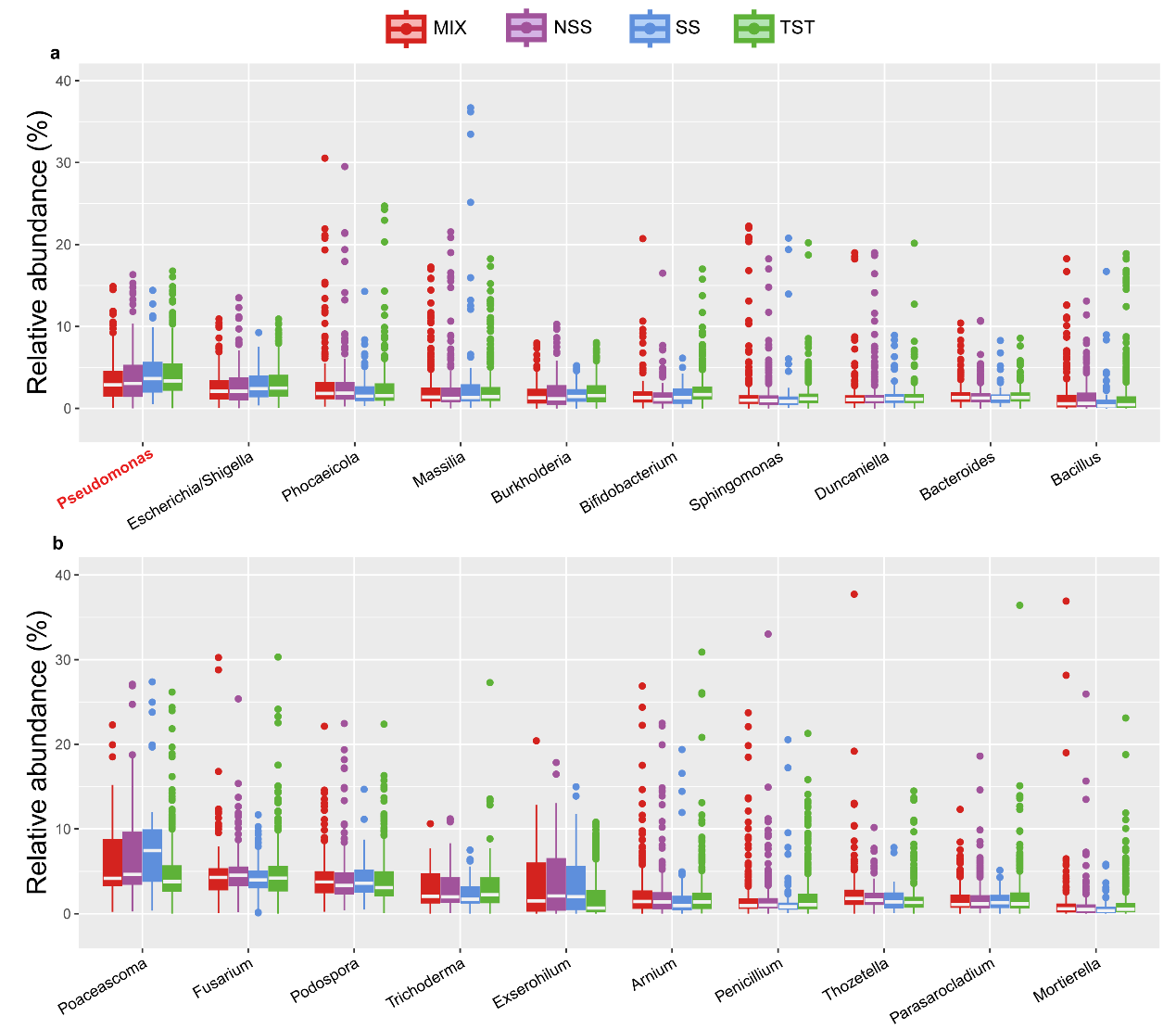


Fig. S6 Relative abundances of the top 10 (a) bacterial and (b) fungal genera. Maize genotypes: MIX, Mixed Subpopulation; NSS, Non-Stiff Stalk; SS, Stiff Stalk; TST, Tropical/Subtropical.


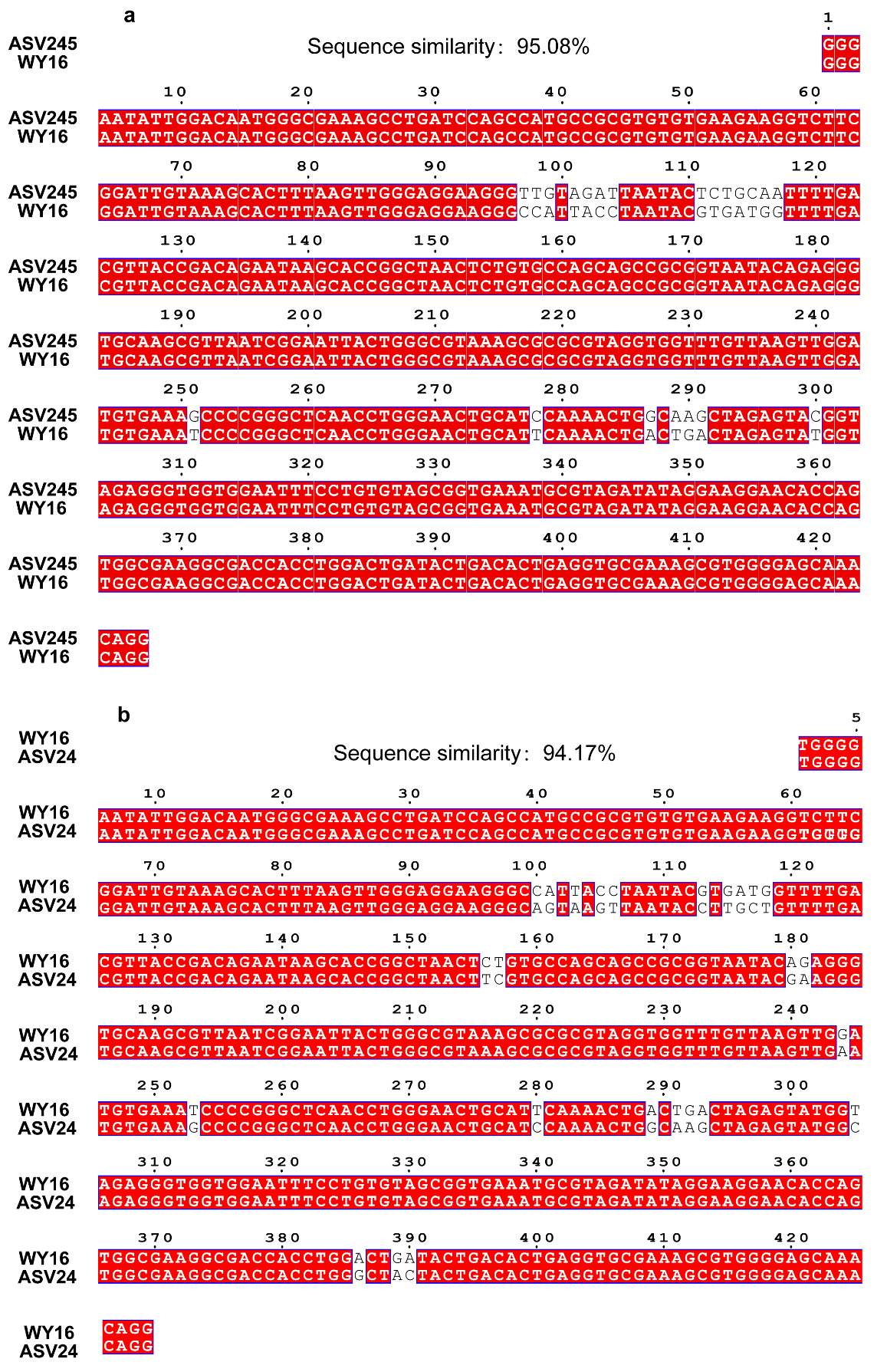


Fig. S7 (a) Sequence alignment between ASV245 and WY16. (b) Sequence alignment between ASV24 and WY16.


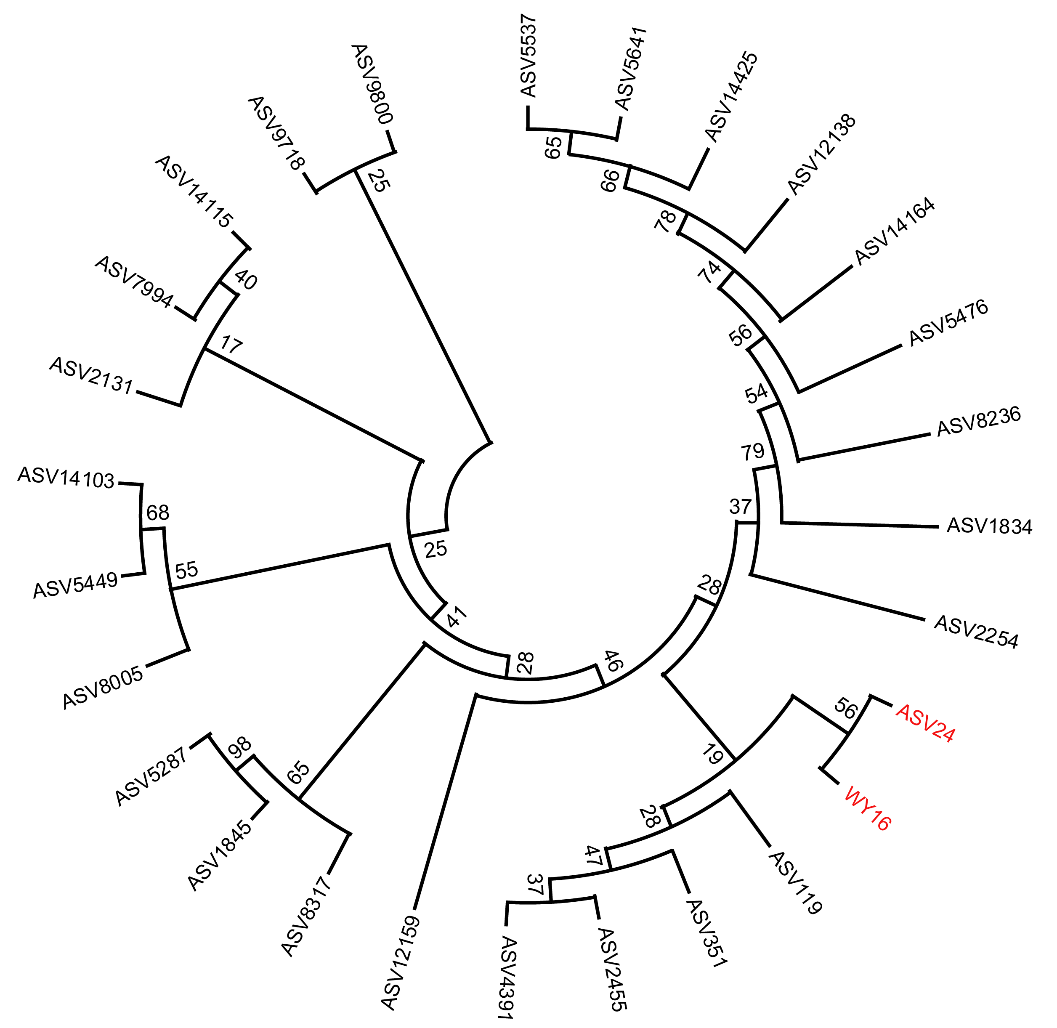


Fig. S8 The phylogenetic tree of WY16 and all ASVs of *Pseudomonas* in pot experiment. WY16 and ASV24 have a closer evolutionary relationship.


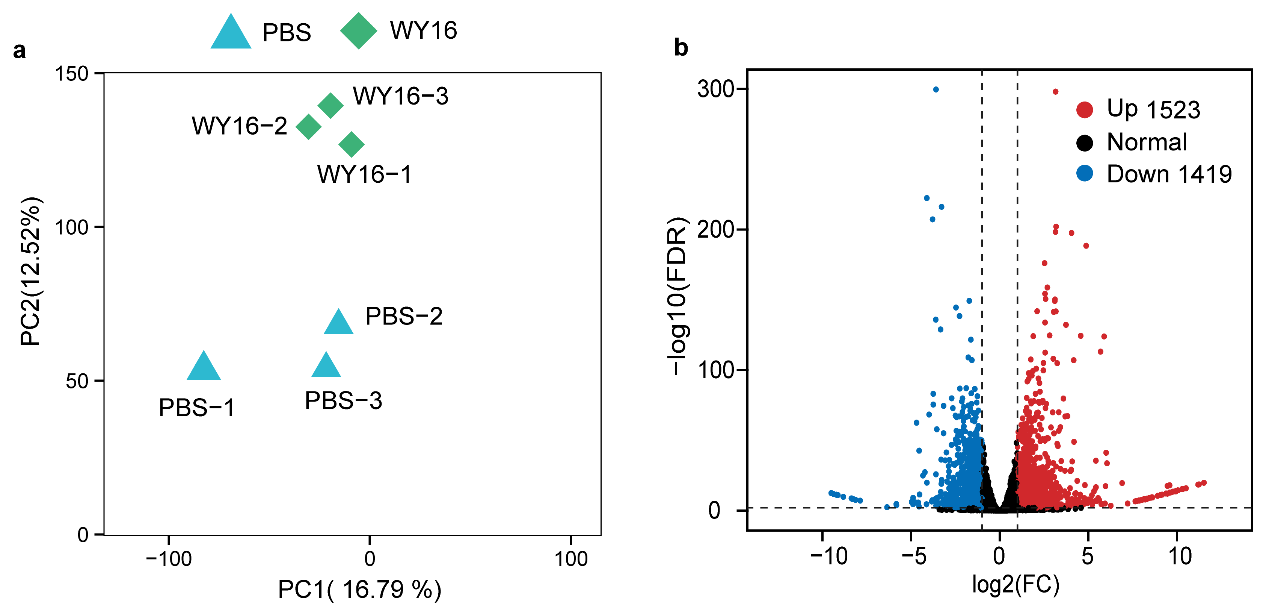
 Fig.S9 Principal component analysis of gene expression (a). Volcano plot of differentially expressed genes (b). The filtering genes: |log2 Fold Change| > 1, and adjusted *P*-value < 0.05.

Fig. S10 Plant hormone signal transduction. The red part indicates that all genes are up-regulated, the green part indicates that all genes are down-regulated, and the blue parts indicate genes that are enriched to both up-regulated and down-regulated at the same time.
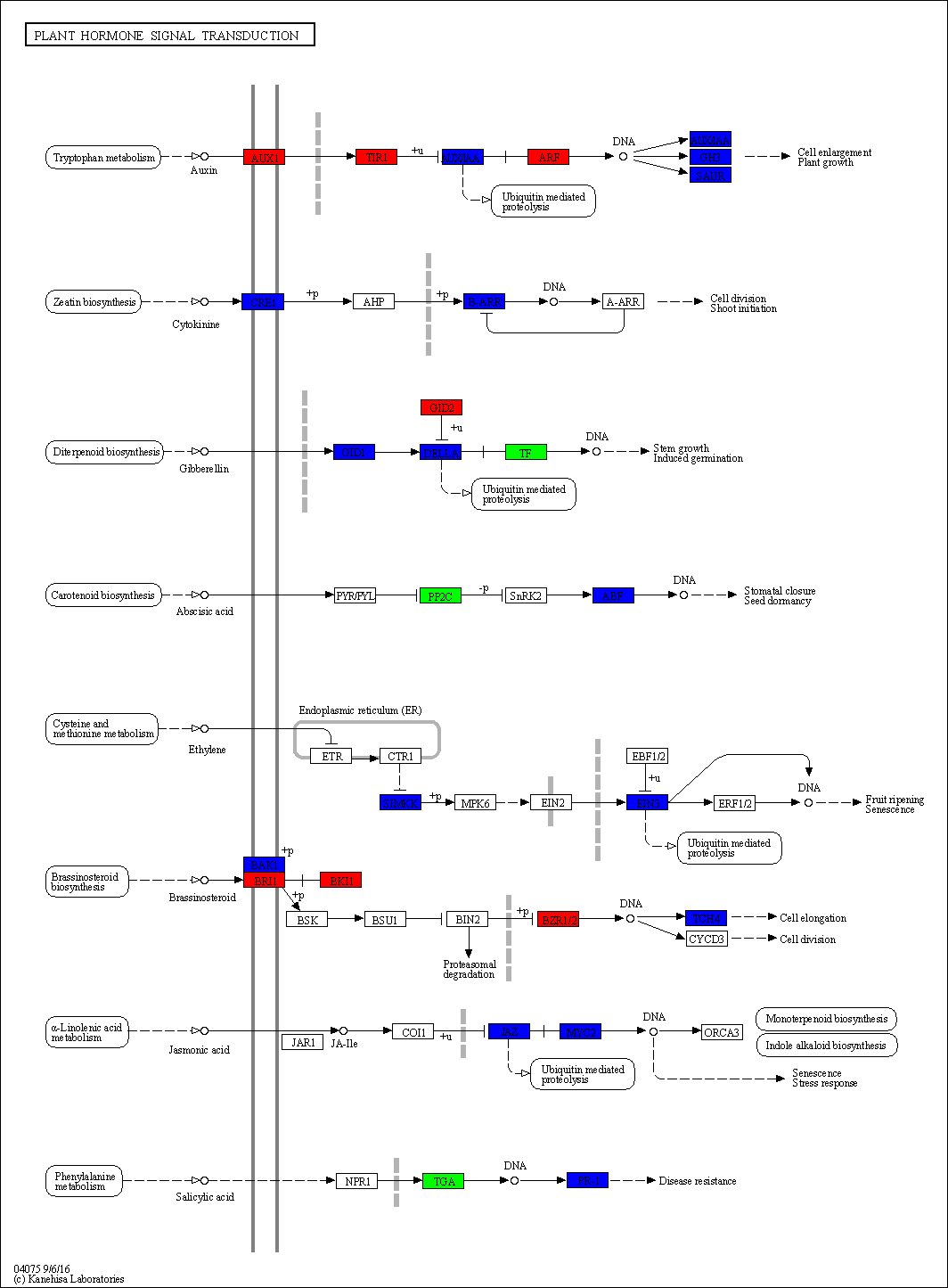

Supplement: Supplementary file 1 — Supplementary Material [file 41522_2025_895_MOESM1_ESM.docx]
